# Natural Polysaccharides for siRNA Delivery: Nanocarriers Based on Chitosan, Hyaluronic Acid, and Their Derivatives

**DOI:** 10.3390/molecules24142570

**Published:** 2019-07-15

**Authors:** Inés Serrano-Sevilla, Álvaro Artiga, Scott G. Mitchell, Laura De Matteis, Jesús M. de la Fuente

**Affiliations:** 1 Instituto de Ciencia de Materiales de Aragón (ICMA), Consejo Superior de Investigaciones Científicas (CSIC)-Universidad de Zaragoza, C/Pedro Cerbuna 12, 50009 Zaragoza, Spain; 2 CIBER-BBN, Instituto de Salud Carlos III, Madrid, Spain; 3 Instituto de Nanociencia de Aragón (INA), Universidad de Zaragoza, C/Mariano Esquillor s/n, 50018 Zaragoza, Spain

**Keywords:** nanotechnology, nanocarriers, polymers, natural polysaccharides, chitosan, hyaluronic acid, small interfering RNA (siRNA) delivery, gene silencing

## Abstract

Natural polysaccharides are frequently used in the design of drug delivery systems due to their biocompatibility, biodegradability, and low toxicity. Moreover, they are diverse in structure, size, and charge, and their chemical functional groups can be easily modified to match the needs of the final application and mode of administration. This review focuses on polysaccharidic nanocarriers based on chitosan and hyaluronic acid for small interfering RNA (siRNA) delivery, which are highly positively and negatively charged, respectively. The key properties, strengths, and drawbacks of each polysaccharide are discussed. In addition, their use as efficient nanodelivery systems for gene silencing applications is put into context using the most recent examples from the literature. The latest advances in this field illustrate effectively how chitosan and hyaluronic acid can be modified or associated with other molecules in order to overcome their limitations to produce optimized siRNA delivery systems with promising *in vitro* and *in vivo* results.

## 1. RNA Interference for Gene Silencing

RNA interference (RNAi) is a biological process that regulates gene expression by sequence-specific gene silencing at post-transcriptional level, preventing the translation of its messenger RNA (mRNA) transcripts into proteins. In 1998, Fire and Mello detected RNA interference for the first time after injecting exogenous double-stranded RNA (dsRNA) in *Caenorhabditis elegans* [1]. Three years later, this RNAi technology was adapted for mammalian cells [2]. Although the RNA interference pathway can be triggered by different dsRNA (small interfering RNA (siRNA), microRNA (miRNA), short hairpin RNA (shRNA), and Piwi-interacting RNA (piRNA) [3,4]), this review will focus solely on research concerning siRNAs, the most common structure currently used in RNAi-based therapeutic formulations.

siRNA is a dsRNA of approximately 21–23 base pairs in length with a characteristic 2-nucleotide overhang at both 3’ ends and complementarity to target mRNA [5]. Its provenance can be endogenous (then denominated endo-siRNA) [6,7], when it is synthesized in the cellular nucleus and later translocated into the cytoplasm, or exogenous, when it is synthesized outside the target cell and delivered into the cytoplasm [8,9]. However, the term siRNA in higher organisms is now commonly used for exogenously synthesized dsRNA [4,10]. Once in the cytoplasm, siRNA participates in the formation of a multi-protein complex, the RNA-induced silencing complex (RISC), which degrades the passenger strand (i.e., sense strand) and incorporates the guide strand (i.e., antisense strand). This strand is used by the RISC complex as a template for the recognition and cleavage of complementary mRNA, preventing the synthesis of the encoded protein to selectively silence gene expression [11,12].

Apart from being a useful technology for studying gene and protein functions, synthetic siRNA has attracted considerable attention as a promising therapeutic tool [13,14,15]. It can be easily designed for the specific silencing of, theoretically, any target gene, including targets traditionally considered to be ‘undruggable’. The high specificity level of siRNA could be enough to target disease-associated mRNA sequences with only one or few mutated nucleotides. Many siRNA-based therapeutics are under development for the treatment of diseases ranging from viral infections, to genetic diseases, and cancers [16,17,18].

**Scheme 1:**
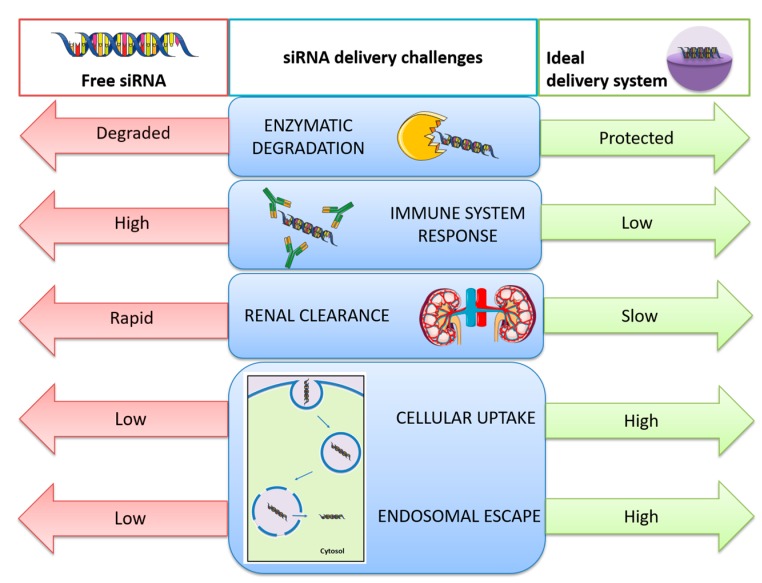
Challenges related with *in vivo* small interfering RNA (siRNA) delivery and solutions provided by the use of nanocarriers to overcome the problems of free siRNA delivery.

Nevertheless, the lack of success of siRNA-based formulations as therapies resides in the challenges facing the *in vivo* delivery of siRNA. Unmodified siRNA is rapidly degraded by endogenous nucleases and induces the innate immune response. Chemical modification of siRNA structure has been found to be a possible strategy that can prevent both enzymatic degradation and immunogenicity, as well as to reduce off-target effects [19]. However, there are some other issues that such modifications cannot solve: the rapid renal clearance after systemic administration due to the relatively small size of siRNA (compared to the effective glomerular pore size), and the inability of RNA to cross the hydrophobic target cell membrane as a result of its hydrophilicity, negative charge, and too high molecular weight. This means that the development of safe and effective delivery systems that can protect siRNA and facilitate its transport into the cytoplasm of target cells can be decisive in obtaining a more successful application of siRNA-based therapies [20]. An ideal carrier for siRNA delivery should possess the following three fundamental attributes: (1) it should efficiently bind siRNA to maintain its stability in physiological media by providing protection against enzymatic degradation; (2) the association with a carrier should help to avoid siRNA clearance by the mononuclear phagocyte system and to enhance the intracellular uptake; and (3) the siRNA–carrier complex should display endosomal escape into the cytosol and produce a sustained release of siRNA without causing toxicity or activating the immune system [21]. The numerous problems related to the *in vivo* delivery of siRNA and solutions potentially provided by nanocarriers are summarized in Scheme 1.

At present, viral-based vectors are being used for siRNA delivery research. However, despite their high efficiency for *in vivo* transfection, their safety remains questionable because of their toxicity, immunogenicity, and inflammatory potential. Furthermore, they have other limitations, such as complex manufacturing processes and low cell specificity [22]. Nevertheless, recent advances in biomaterial and nanotechnology sciences have led to the development of non-viral nanocarriers as alternatives for siRNA delivery. Nanostructured delivery systems provide unique advantages, like protection from premature degradation and improved interaction with the biological environment. They also offer the possibility to enhance the absorption into a selected tissue, extend siRNA retention time, and improve cellular internalization [23].

Nanostructured siRNA delivery systems include a wide variety of nanocarriers, defined as submicron-sized colloidal systems (with a size below 1 µm) [24], such as inorganic nanoparticles, lipidic, and polymeric nanocarriers [25]. At present, lipid-based siRNA delivery systems are clearly the most studied non-viral vectors, with several formulations in late stage clinical trials, such as lumasiran and givosiran [26], as well as patisiran (Onpattro, Alnylam Pharmaceuticals): the first-ever siRNA therapy approved by the FDA on 10 August 2018 [27,28]. Far below in number, also some polymer-based siRNA delivery systems have reached clinical trials, such as siG12D-LODER [29], although none have been approved yet [30]. Polymeric nanocarriers can be prepared from different natural or synthetic polymers. Natural polymers have some advantages over their synthetic counterparts: they are nontoxic, cheaper, and naturally available [31] and, in contrast to inorganic nanoparticles and some synthetic polymers, they are usually biodegradable. Among polymer-based nanocarriers, those obtained from naturally occurring polysaccharides are worthy of a special mention since they are commonly considered to be highly biocompatible and non-immunogenic, placing them in the fast track as potentially commercial therapies.

## 2. Use of Natural Polysaccharides as Nanocarriers for Gene Silencing

Natural polysaccharides can be obtained from animals (e.g., chitosan, hyaluronic acid), plants (e.g., starch, cellulose, cyclodextrins, pectin), algae (e.g., alginate), or bacteria (e.g., dextran) [31,32], and they have a wide range of applications, from agriculture and food industries to cosmetics, as well as the pharmaceutical and biomedical fields [33]. Polysaccharides are formed by monosaccharides forming carbohydrate structures that can be neutral or charged, linear or branched, and with varying degree of hydrophilicity [32,34].

The widespread use of natural polysaccharides in medicine for drug delivery is their biocompatibility, biodegradability, and low toxicity [12]. They have also been employed in combination with synthetic materials as a strategy to mitigate the toxicity of the latter [32]. Their structural diversity allows choosing the most suitable polysaccharide for each case depending on the needs of the final application, and more importantly, their functional properties can also be tailored and optimized by chemical modifications to their functional groups [31,34].

Many natural polysaccharides possess bioadhesive properties thanks to the presence of specific functional groups, making them useful starting materials for nanocarrier design with improved residence time, especially for intestinal and pulmonary applications. The presence of such groups also allows specific modification of the polymer structure aimed to improve their delivery or targeting ability. Furthermore, natural polysaccharides can be neutral (e.g., cellulose, pullulan, starch, dextran), negatively (e.g., hyaluronic acid, alginate, chondroitin sulphate), or positively charged (e.g., chitosan).

**Figure 1:**
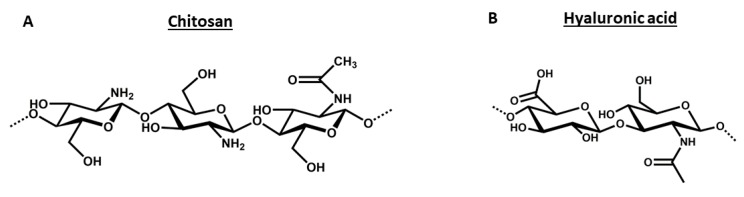
Chemical structures of chitosan (**A**) and hyaluronic acid (**B**).

Of all the polysaccharides used for siRNA-based therapies, we have specifically selected chitosan and hyaluronic acid as the subjects of this review in order to highlight their applicable properties and characteristic features that make them so relevant for siRNA delivery. Although they are both mucoadhesive polymers, they possess opposite electrostatic charges, strongly influencing their use for nucleic acid delivery. This important difference derives from their chemical structure, which offers positively charged amino groups in the glucosamine monomers of chitosan or negatively charged carboxylic groups in the glucuronic acid units of hyaluronic acid. The structures of both polymers are shown in Figure 1.

Cationic polysaccharides, such as chitosan, can electrostatically interact with siRNA to form stable, positively charged polyplexes, while neutral and anionic polysaccharides often require the presence of other components to favor a more efficient interactions with siRNA [35]. On the other hand, the association of siRNA with anionic polysaccharides like hyaluronic acid can improve bioavailability by avoiding siRNA excretion through the glomerular capillary wall. In fact, negatively charged species are “less filterable” because of the repulsion force that is established with the negative charge of the glomerular membrane [36]. Nevertheless, the most efficient intracellular delivery of nucleic acids is apparently achieved by positively charged nanocarriers. This can be explained by two factors. Firstly, the efficient physical interaction with the negatively charged nucleic acid protects them from nuclease degradation. Secondly, they provide a net positive charge, which enhances the interaction of the complex with the anionic surface of cells [37].

**Figure 2:**
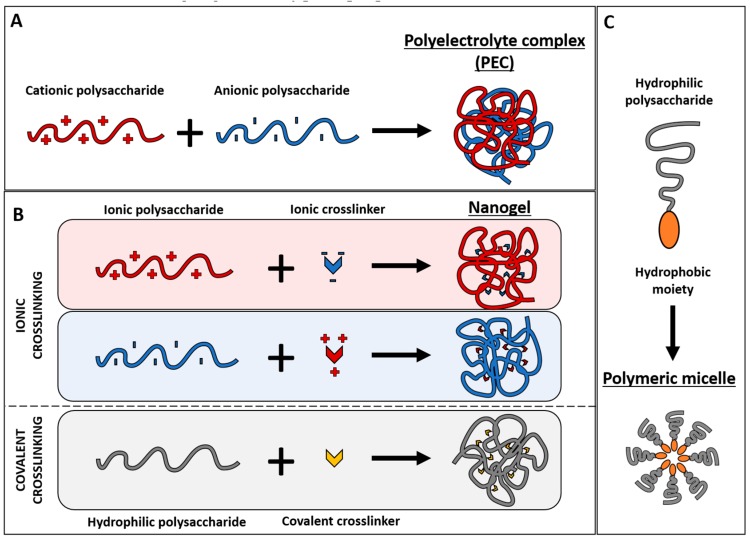
Types of polysaccharide-based nanoparticles depending on their components, the interactions between them and their final structure: polyelectrolyte complex (**A**); nanogel (**B**); and polymeric micelle (**C**).

Polysaccharides and their derivatives can be used to obtain different types of nanocarriers. This review will mainly focus on siRNA delivery systems depending only on the interaction of siRNA with a polysaccharide, specifically chitosan or hyaluronic acid or derivatives of these polysaccharides, without mentioning systems that also include lipids because it would enhance enormously the complexity of the system complicating the extraction of general conclusions about the polymers of interest. Most nanocarriers reported in the examples included in the following sections correspond to polyelectrolyte complex nanoparticles, nanogels, and polymeric micelles. Such polysaccharide-based nanoparticles are summarized in Figure 2, and described below to define their structures and highlight their typical properties.

### 2.1. Polyelectrolyte Complexes

In general, polyelectrolyte complexes (PECs) are spontaneously formed when two polymers of opposite charge are mixed, forming a complex where macromolecules are bound together mainly by electrostatic interactions [38]. Furthermore, the term “nanoplex” is widely used in the literature to designate a complex formed between a positively charged nanoparticle and a nucleic acid, either DNA or RNA [15,34,39,40,41]. If the positively charged component is based on a cationic polymer, such as chitosan, the nanoplexes can be defined also as polyplexes [42,43].

It is well recognized that PECs are mainly spherical and consist of a neutral core, containing stoichiometric mixtures of cationic and anionic polymers, surrounded by an excess of one of the polyelectrolytes that allow the formation of a charged stabilizing shell [42,44].

Since self-assembly of PECs is a reversible process, their integrity in the presence of other charged species can be compromised due to electrostatic shielding and polyion exchange between the PEC and the medium. Therefore, the stability of PECs can be seriously affected in biological media, where there are charged proteins and high concentrations of small electrolytes. A strategy to overcome this problem is to enhance the stability of PECs by covalently cross-linking the complexed polyelectrolytes, preventing the detachment of polymer chains from the PEC [44]. PEC-based nanoparticles are attractive since they are easy to prepare, cost-effective, and because the reversibility of the electrostatic interactions offers special tunability of the carrier’s behavior while maintaining the integrity of the complexes under appropriate conditions [45].

### 2.2. Nanogels

Nanogels (or hydrogel nanoparticles) consist of three-dimensional, highly hydrated networks of polymeric chains electrostatically or covalently cross-linked [45,46,47]. Although they can be synthesized by different methods, the ionotropic gelation method is one of the most commonly used to obtain nanocarriers for nanomedicine [46]. This method is based on the establishment of electrostatic interactions between the charged polymer chains and an oppositely charged appropriate ion or polyion, which acts as ionic cross-linker. The most used ionic cross-linkers are low molecular weight ions, such as sulphate salts and calcium chloride, and polyionic cross-linkers such as sodium tripolyphosphate (TPP). Ionotropic gelation offers advantages including mild preparation conditions and simple experimental procedures. On the other hand, covalent crosslinking can be used to enhance the colloidal stability of nanogels under physiological environments by increasing their rigidity and compaction thanks to the strong covalent bonds. The most frequently employed covalent crosslinkers are glutaraldehyde and formaldehyde, but their use in drug delivery is limited due to their high cytotoxicity [45,47].

Nucleic acids can be loaded into nanogels once these are already formed (post-synthetic loading), leading to an adsorption of the negatively charged molecule on the positively charged surface of the hydrogel nanoparticles, or during their synthesis to be entrapped inside the nanogel. This second strategy usually leads to higher encapsulation efficiencies and improved protection of the nucleic acid from degradation, although sometimes it can lead to a more difficult release [48]. When positively charged nanogels are loaded with nucleic acids via electrostatic interactions, it is important to note that, by definition, the obtained nanosystem could also be considered to be a polyelectrolyte complex (PEC).

### 2.3. Polymeric Micelles

Micelles are self-assembled, nanosized colloidal particles with a hydrophobic core and a hydrophilic shell. The abundant functional groups along the hydrophilic polysaccharide backbone facilitate attachment of hydrophobic moieties, leading to amphiphilic copolymers [49]. Amphiphilic polysaccharides self-assemble to form micelle nanoparticles upon contact with an aqueous environment in response to a shift in the hydrophilic/hydrophobic balance [36]. While the hydrophobic micelle core provides stability and enables the loading of hydrophobic drugs, the hydrophilic shell can be modified with cations that interact electrostatically with siRNA, which makes micelles very suitable for combined delivery of siRNAs and drugs [50].

## 3. Chitosan for siRNA Delivery

Chitosan is a biopolymer derived from the deacetylation of chitin, which can be extracted from the exoskeleton of crustaceans (crabs, shrimp, krill, etc.), insects, and from cell walls of fungi [51]. It is a linear polymer that consists of repeating units of N-acetylglucosamine and glucosamine monomers linked by β(1→4) glycosidic bonds (Figure 1). Its degree of deacetylation and molecular weight are both known to significantly affect the properties of derived materials [42,52]. The amino and hydroxyl groups present in the chitosan chains facilitate the chemical modification, and so the tunability of its structure and functional properties [22]. These primary amino groups are also responsible for the positive charge that chitosan exhibits below pH 6, making it useful for applications under slightly acidic conditions, such as tumoral extracellular environments. However, at the physiological pH of blood the charge of chitosan is reduced, leading to a loss of both efficacy in siRNA complexation and nanoparticle stability. Together with added problems of interaction with serum proteins, this stability issue can represent a major problem for *in vivo* gene silencing upon systemic delivery [53].

Nevertheless, due to their natural nautre, positively charged polysaccharides have led to the extensive use of chitosan for nucleic acid delivery [49]. One of its main advantages is that its cationic nature enhances the electrostatic interaction with the negatively charged RNA, favoring the formation of stable polyplexes. The siRNA binding ability of chitosan stands out over the rest of natural polysaccharides, which are usually neutral or negatively charged. This is the case for polyanion hyaluronic acid, which must be chemically modified or associated to a cationic component to favor its use in nucleic acid delivery (the strategies developed for this purpose are thoroughly described in the Section 4.2).

In addition to the previously mentioned advantageous properties of being a natural polymer, chitosan has some special features rendering it as highly relevant for therapeutic applications. Its low immunogenicity [51] avoids triggering an immune response that could rapidly remove foreign particles from the body. It also has excellent mucoadhesive properties mainly due to the interactions of its protonated amino groups with the negatively charged mucus layer, which may prolong the contact time between the nanocarrier and the absorption site [54]. Furthermore, chitosan is able to open the intercellular tight junctions of the intestinal epithelium in a transitory and reversible manner, thus increasing the epithelial permeability through paracellular absorption and accelerating the transport of the therapeutic agent [55,56,57]. These properties are the main reason for the frequent use of chitosan in the development of mucosal drug delivery systems and tissue engineering [31,32]. Chitosan is currently approved by the American Food and Drug Administration (FDA) as a wound dressing material and also as a dietary component [54,58].

### 3.1. Factors Affecting Gene Silencing Efficiency

Many studies have been performed in order to identify fundamental chitosan molecular properties favoring efficient siRNA delivery and gene silencing. For example, a high degree of deacetylation (over 80%) seems to be the best to increase the polymer’s positive charge and enable a greater siRNA loading capacity [21].

Among all the parameters affecting the gene silencing efficiency of chitosan nanoparticles, the most investigated is the molecular weight of chitosan. Some authors found an increasing gene silencing efficacy with polyplexes obtained using high molecular weight chitosan [59,60], others achieved a better efficacy using lower molecular weight chitosan [61], while others found no obvious correlation [62]. Due to the controversial results from different research groups, it is difficult to extract a general conclusion from the literature about the influence of the molecular weight of chitosan. A robust and exhaustive study to evaluate the entire range of molecular weights under the same conditions should be performed.

On the other hand, it has been observed that the choice of a specific preparation method to obtain the siRNA-loaded nanocarrier plays an important role on the interference effect. Katas and co-workers realized that the siRNA delivery was more efficient when siRNA was complexed with chitosan by ionic gelation, than by simple complexation or adsorption [62]. Moreover, the stability and efficacy of siRNA-loaded chitosan nanoparticles prepared by ionic gelation is highly dependent on the cross-linker used. Raja et al. showed that sodium tripolyphosphate (TPP) produced the chitosan nanocarrier with the lower size and the most stable system, with high siRNA binding efficiencies, compared to dextran sulphate (DS) and poly-d-glutamic acid (PGA) [63].

Chitosan amine to siRNA phosphate ratio (N:P ratio) is also an important parameter to take into account since it affects several physicochemical properties of the obtained polyplexes. An excess of chitosan over siRNA is needed to form a compact polyelectrolyte complex. As a high N:P ratio allows a stronger binding of siRNA, most literature describes the use of high N:P ratios, ranging from 20 up to 200 in some cases [42,59,60,62]. Moreover, the N:P ratio strongly influences the nanocarrier’s zeta potential. Highly positive surface potential favors the colloidal stability of non-sterically stabilized polyplexes and enhances the interactions with the negatively charged cell membrane, leading to high cellular uptake and transfection efficiency *in vitro* [42]. However, despite the higher stability provided by the high N:P ratio, excess chitosan can also lead to higher cytotoxicity, which decreases the upper limit of siRNA dosage [53]. In contrast, some nanocarriers synthesized using low N:P ratios (4–10) showed *in vitro* efficient uptake and gene silencing, reaching levels similar to those obtained using Lipofectamine® [61,64,65]. With regards to adverse effects, the low N:P ratio nanocarriers presented minimal impact on cell viability and could be an interesting alternative to so far reported chitosan polyplexes that used chitosan excess to achieve similar bioactivity. However, *in vivo* experiments are needed to evaluate if this reduced amount of chitosan with respect to that of siRNA provides enough stability to the delivery system at physiological conditions. It has been reported that the N:P ratio can also affect the mucosal penetrability and the biodistribution pattern after oral administration [66].

### 3.2. Applications of Native Chitosan-Based Nanocarriers

The interesting qualities of chitosan, such as biocompatibility, mucoadhesive properties, rapid cell internalization, and high siRNA binding capacity, have led to the development in recent years of an array of siRNA-loaded chitosan nanocarriers for very different applications. Here, we report some of the most interesting examples found in the literature on siRNA delivery systems based on native chitosan.

A possible therapeutic alternative to prevent the internalization of Hepatitis C virus into liver cells in the disease’s early stages has been addressed to inhibit the expression of the scavenger receptor class B type 1 (SR-B1). siRNA-loaded chitosan nanocarriers were able to silence the SR-B1 after 96 hours of incubation with HepG2 cells [67].

Another study, aimed to inhibit cancer angiogenesis, described the feasibility of using chitosan nanocarriers for successful siRNA delivery to finally reduce the vascular endothelial growth factor (VEGF) levels in a mouse melanoma model *in vitro* (silencing efficiency of 40%) [68].

An interesting application in the field of gene silencing involves the local delivery of siRNA to the lungs through nebulization. Nebulization of biopharmaceuticals in solution may result in their degradation and their association with carrier particles can improve their stability [69]. In the study of Sharma and co-workers, siRNA-loaded chitosan nanocarriers were stable at the pH of lung airways (i.e., pH 6.5) and were able to protect the siRNA against the physical stress from nebulization process [70].

The first use of siRNA nanotherapeutics in biocompatible implants for the regeneration of the nervous system involved chitosan nanocarriers [71,72]. Resorbable microfilaments of block-copolymer used as nerve implants were loaded with siRNA-loaded chitosan nanocarriers to promote nerve regeneration and allow local delivery of nanotherapeutics. These nanoparticles were rapidly internalized by PC12 cells (a neuronal cell model), without affecting cell viability. Target mRNA of protein RhoA, a protein involved in the inhibition of nerve tissue regeneration, was successfully reduced by 65–75%, and neurite outgrowth was enhanced even in an inhibitory environment [71].

Non-invasive RNAi methodology using chitosan/dsRNA self-assembled nanocarriers were reported to successfully mediate gene silencing in mosquitos through larval feeding. Although mosquitos transmit many of the world’s deadliest diseases, mosquito developmental biology remains understudied. This research illustrated the potential for using chitosan/siRNA nanocarriers as an effective mean of targeting genes during post-embryonic development. Such studies will be fundamental for the screening of gene functions and for identification of key developmental genes for the design of novel strategies to prevent, diagnose, and treat mosquito-borne illnesses on human health, like malaria, dengue, or yellow fever [73,74].

### 3.3. Modifications to Improve the Efficiency of Chitosan-Based siRNA Nanocarriers

Despite the broad range of applications of siRNA delivery systems based on native chitosan, most successful studies in the therapeutic field have been performed only under *in vitro* conditions, with a few limited *in vivo* studies having achieved results promising enough to enter the clinical phases [75,76].

This huge development and evaluation of chitosan-based siRNA delivery systems has allowed the identification of several limitations. For example, their poor stability at physiological pH, due to the loss of positive charge, their weak buffering capacity, which is insufficient to ensure the endosomal escape, and their lack of cell specificity to enhance their preferential internalization in the target cells over the non-targeted ones [77]. However, the understanding of these limitations, together with the perspective of the advantages of the use of this natural polymer (e.g., biocompatibility, biodegradability, low cytotoxicity, and mucoadhesive properties) has encouraged research into alternative derivatives of chitosan that endow the delivery systems with improved or new properties [22,53]. Table 1 includes the most relevant examples of chitosan modifications that have been recently developed to increase the efficiency of siRNA delivery.

**Figure 3:**
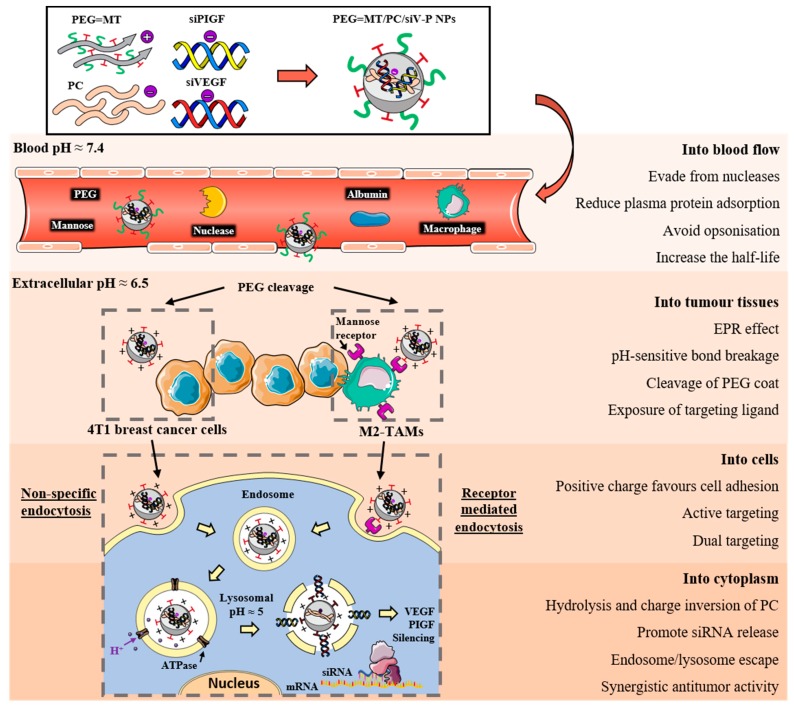
Schematic illustrations of the preparation of modified chitosan nanocarriers and their antitumor efficacies overcoming the systemic and intracellular delivery barriers for the synergistic delivery of two siRNAs. The nanoparticles were synthesized using polyethylene glycol (PEG) and mannose doubly modified trimethyl chitosan (PEG = MT) along with citraconic anhydride grafted poly (allylamine hydrochloride) (PC). Adapted from [90] with permission from Elsevier.

A graphical example of how the multi-functionalization of chitosan nanoparticles can improve siRNA delivery from the blood to the cell cytoplasm is shown in Figure 3 [90]. In this recent work, Song and co-workers developed polyethylene glycol (PEG) and mannose doubly modified trimethyl chitosan (PEG = MT) along with citraconic anhydride grafted poly (allylamine hydrochloride) (PC) nanoparticles. Each of these modifications played a role to overcome the systemic and intracellular delivery obstacles: PEG stabilized the system and prolonged blood circulation, achieving a high accumulation in tumour tissues by passive targeting; mannose ligand enhanced the internalization in macrophages by active targeting; and PC conferred pH-sensitivity for an effective endosomal escape. However, although the combination of all these modifications ameliorates the chitosan nanocarrier properties for siRNA delivery, it requires a very refined optimization process that a simpler system would not need. Furthermore, very complex systems lead to manufacturing difficulties when adapting their synthesis to high-scale production, which is one of the major issues that is currently delaying the clinical translation of very efficient delivery systems [107].

#### 3.3.1. Chitosan Solubility and Delivery System Stability

Although chitosan is water soluble in slightly acidic conditions due to the protonation of its amino groups, at physiological pH these groups become deprotonated, leading to the decrease of both polymer solubility and siRNA binding ability in biological media, which compromises the stability of the nanoparticles and ultimately the biological activity of the siRNA [80].

The inclusion of anionic polysaccharides, like hyaluronic acid [103], or anionic derivatives of dextran [17,93], to chitosan nanocarriers has proved in some cases to increase the stability of the whole structure and to allow efficient siRNA delivery.

The chemical grafting with polyethylene glycol (PEG) is another strategy widely used to improve the physicochemical characteristics of chitosan-based nanocarriers in biological medium [80,108,109,110,111]. It prevents chitosan–siRNA complexes aggregation and enhances their steric stabilization, diminishing the unspecific interactions with serum proteins, and thus prolonging their *in vivo* circulation time [78,100,112]. PEG can also be used as a spacer between the nanocarrier surface and a specific moiety or molecule linked on nanocarrier surface for a targeted siRNA delivery [78,79,82,91,100,102,105].

Rudzinski and co-workers observed that at a low density of coating, the presence of PEG on chitosan nanocarriers’ surface did not affect cellular uptake, and both pegylated and non-pegylated chitosan nanoparticles were as effective as Lipofectamine® [98]. However, there are some studies affirming that the knockdown efficiency is consistently reduced after increasing PEG’s density [100]. This effect is attributed to a reduced surface potential of the chitosan nanocarrier, which diminishes cellular uptake. To address this issue, Corbet and colleagues opted for a non-covalent attachment of PEG chains, which are removed with time. In this way, PEG provided a sheddable stealth protection compatible with efficient siRNA delivery in cancerous mice [82].

The addition of specific chemical groups, such as acyl [81,83,113] and carboxymethyl groups, [99,114], into chitosan’s structure to improve its solubility in aqueous media at neutral and alkaline pH has been explored to improve siRNA delivery. Furthermore, the partial quaternization of chitosan amino groups produces trimethylchitosan (TMC), which has stable positive charges, making it soluble over a wider pH range and concurrently conferring enhanced mucoadhesive properties, although it may negatively affect its buffering capacity and increase cytotoxicity [77,84,92,104,115].

**Figure 4:**
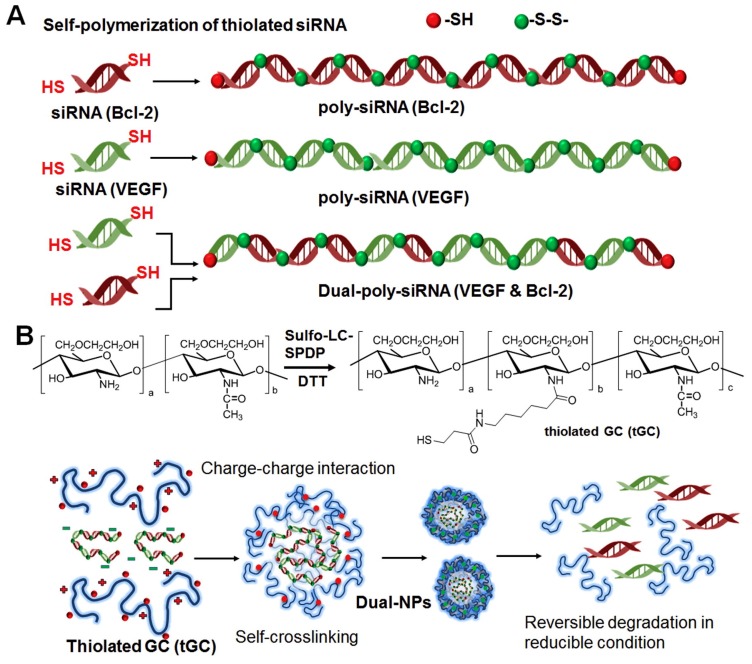
Schematic illustration of co-delivery of VEGF and Bcl-2 siRNAs using the dual-nanoparticle (dual-NP) system. Dual-poly-siRNA (**A**) was incorporated in the thiolated glycol chitosan (tGC) to form nanoparticle structures (dual-NPs) through the charge-charge interaction and chemical crosslinking (**B**). Adapted from [85] with permission from Elsevier.

Some authors used low molecular weight chitosan to improve the solubility due to the reduced inter-chain interactions between short chains [77]. While others used ionic or covalent cross-linking of chitosan chains to make the resulting nanocarriers more compact and thus more resistant to polyanion competition *in vivo,* avoiding the siRNA displacement by other negatively charged molecules present in physiological tissues [84,85,86,104,116,117]. Lee and colleagues created a dual-nanocarrier system for the *in vivo* co-delivery of different siRNA. They demonstrated that self-crosslinking of thiol groups in glycol chitosan polymers plays an essential role for obtaining condensed nanoparticles since unmodified polymer without thiol groups could not form stable complexes under the applied conditions [85]. The advantage of establishing disulphide bonds between thiol groups for polymer cross-linking consists in the fact that they can be reduced in the intracellular reductive conditions, leading to the release of the siRNA molecules (Figure 4) [85,86].

Covalent conjugation of chitosan with biocompatible cationic peptides such as protamine [77], poly-l-lysine [81], or nonaarginine [106] has been used as strategy to increase affinity for siRNA and so to improve the stability of the siRNA/chitosan complexes in physiological conditions. At the same time, cationic peptides can promote cell interaction and uptake due to the increased positive surface charge of the nanocarrier and some, such as histidine-based peptides, also exhibit high buffering capacity, enhancing endosomal escape.

#### 3.3.2. Cellular Uptake and Endosomal Escape

Cells generally tend to internalize nanoparticles into endocytic vesicles called endosomes, where pH progressively decreases meaning that nanocarriers and their cargos are prone to degradation, rendering them useless [118]. Various approaches have been used to promote early endosomal escape of siRNA, for example the employment of high buffering polymers or cell-penetrating peptides.

Some cationic polymers, such as polyethylenimine (PEI) and polyamidoamine (PAMAM) dendrimers, possess high buffering capacity and so they have the ability to disrupt the endosomal membrane and release the siRNA into the cytosol, thus preventing their premature degradation. This effect of pH buffering is also known as the “proton sponge” effect. The buffering capacity of the cationic polymer has two consequences: first, the inhibition of the activity of lysosomal nucleases; second, the change in the osmolarity of acidic vesicles, which results in endosomal swelling and rupture [119]. Although there are some authors supporting the fact that chitosan presents a high buffering capacity in the endosomal pH range [77,120], others tried to improve this effect by grafting chitosan with pH sensitive molecules that have higher buffering ability. The introduction of imidazole moieties into the chitosan backbone has proven to be effective in promoting the escape of the nanoparticles from the endocytic pathway [92,121]. Recently, Xiao and co-workers formed chitosan-based nanocarriers upon complexion of siRNA with urocanic acid-modified chitosan, which contains imidazole groups. Although these nanocarriers exhibited weaker intracellular uptake in Raw 264.7 macrophages, an improved gene silencing effect was observed, and it has been attributed to their increased capacities for siRNA condensation and endosome/lysosome escape [101]. The amine-rich polymer polyethylenimine (PEI) has also been associated with endosomal escape capacities [17,99]. Its use in combination with chitosan has also led to high gene silencing efficacy [102,103].

On the other hand, cell-penetrating peptides (CPPs) are used in conjugation with chitosan-based nanocarriers to improve the siRNA-mediated gene silencing. CPPs, such as the peptide called Trans Activator of Transcription (TAT), nonaarginine [106], and poly(histidine-arginine)_6_ (H6R6) [88], have been shown to destabilize membrane bilayers in cells, although the exact mechanism remains controversial. Therefore, apart from enhancing the penetration of macromolecules across the plasmatic membrane, they are also used for promoting endosomal release [88,105,106,119,122].

#### 3.3.3. Cell Targeting Specificity and Biodistribution

In contrast to other polysaccharides that exhibit targeting ability similar to hyaluronic acid, chitosan polyplexes are mostly internalized in cells via a non-specific adsorptive endocytosis mechanism, due to the lack of specific cell receptors [22]. Consequently, a number of different chitosan-based nanocarriers have been functionalized with a range of specific ligands to achieve targeted siRNA delivery [90,91,123].

By targeting normal enterocytes and M cells that express mannose receptors, mannose-modified trimethyl chitosan-cysteine nanocarriers, notably improved intestinal absorption of siRNA in rats compared to the non-targeted nanocarriers. They were then internalized into gut-associated macrophages as well as systemic macrophages via mannose targeting, where they triggered effective siRNA transfection and TNF-α knockdown, protecting rats from acute hepatic injury [84].

Peptides have also been frequently used to enhance specificity in cell targeting. Nascimento and co-workers functionalized their chitosan nanocarrier with a peptide targeting the epidermal growth factor receptor (overexpressed in cancer cells). It exhibited a six-fold higher cancer-targeting efficiency compared to the non-targeted nanocarrier in lung cancer xenograft models [78]. The Arg-Gly-Asp tripeptide motif (RGD peptide) and peptidomimetics (RGDp) are known to preferentially bind the αvβ3 integrin, which is a cell surface receptor overexpressed both on some cancer cell types and on angiogenic endothelial cells [82]. Corbet and colleagues identified an optimal combination of the RGDp chemical structure, linker nature, and grafting method that increased siRNA delivery to the tumour and led to a significant inhibition of cancer growth upon both peritumoral and intravenous administration in mice [82]. Ragelle and co-workers also grafted RGD and RGDp on their PEGylated chitosan–PEI hybrid nanocarriers and demonstrated that the cellular internalization of targeted nanocarriers was highly dependent on the number of ligand molecules on the surface [102].

In a recent study, improved targeting that involved the use of a protein as targeting element was reported. Low-density lipoprotein (LDL), whose receptor is highly expressed in the cancer cell’s membrane, was used to functionalize N-succinyl chitosan lipoic acid micelles [83]. Attempts on active cell targeting have also been done by the use of antibodies conjugated to the nanoparticles [89,124]. Gao and co-workers developed siRNA/chitosan nanocomplexes conjugated with IgG antibody moieties to facilitate receptor-mediated phagocytosis via the Fc receptors expressed on M1 macrophages. Although the treatment with the IgG-conjugated nanocarrier achieved a significant gene silencing effect, gene silencing was also achieved when using the nanocarrier without the antibody (albeit with lower efficacy). The authors hypothesized that chitosan may be binding to other receptors expressed by macrophages, such as the mannose receptors [89].

#### 3.3.4. Co-Delivery of siRNA and Pharmaceuticals

Certain illnesses, such as cancer, are particularly challenging to treat due to drug resistance and the complex tumour microenvironment. The combination of traditional chemotherapy with gene therapy could significantly augment drug efficacy and allow lower doses, alleviating their secondary effects and overcoming drug resistance. However, siRNA and chemotherapeutics exhibit quite different physicochemical properties, which results in different pharmacokinetics and biodistribution *in vivo*. This makes the development of delivery systems able to carry both siRNA and drug to the same target site at the appropriate time necessary [79,86].

**Figure 5:**
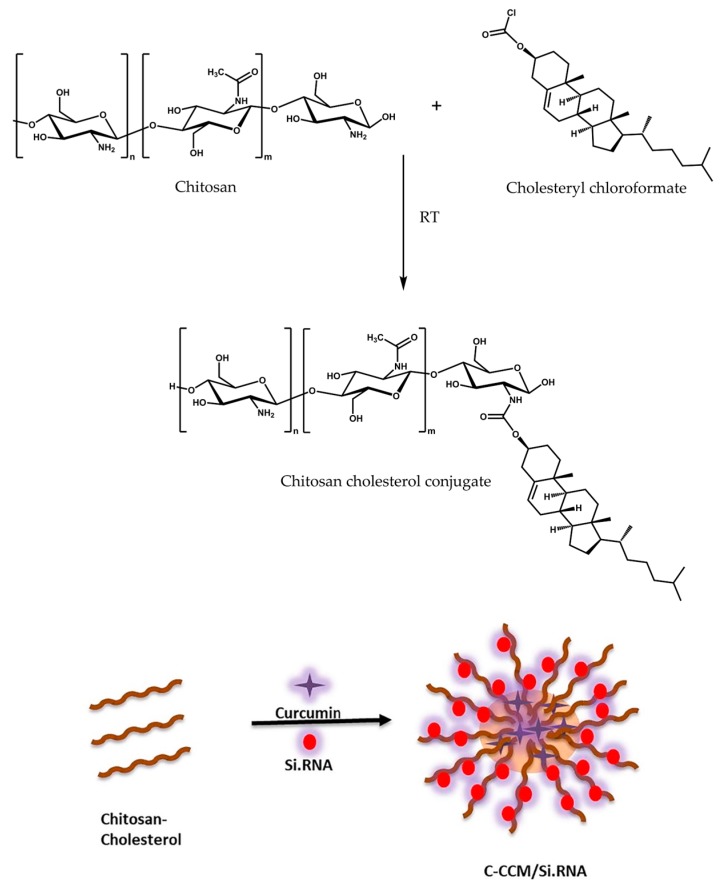
Structure of a cholesterol-grafted chitosan micelle loaded with siRNA and curcumin for co-delivery. Adapted from [96] with permission from Elsevier.

Chitosan can be modified with hydrophobic moieties for the formation of a polymeric micelle where hydrophobic drugs can be encapsulated into the core. The hydrophobic moieties most frequently used are lipophilic acids, such as lipoic acid [83], cholanic acid [86] or palmitic acid [81], and cholesterol [96]. Figure 5 shows the structure of a cholesterol-grafted chitosan micelle developed for drug–siRNA co-delivery to cancer cells [96]. siRNA is complexed within the chitosan hydrophilic shell while curcumin is loaded into the hydrophobic core of the nanocarrier.

## 4. Hyaluronic Acid for siRNA Delivery

Hyaluronic acid (HA), also called hyaluronan, is a linear polysaccharide formed by glucuronic acid and N-acetylglucosamine units linked via alternating β-1,4 and β-1,3 glycosidic bonds (Figure 1) [52]. Hyaluronic acid is present in all vertebrates and some microorganisms. It is typically extracted from bovine vitreous humor, rooster comb, and some bacteria for biotechnological use [125]. As a free polymer, hyaluronic acid plays different biological roles in living organisms [126]. It is implicated in cell growth and migration, embryonic development, healing of tissue injuries, cancer development, and regulation of inflammation [127]. Hyaluronic acid has also been employed in biomedical and cosmetic applications in very different forms such as coatings, matrices, and hydrogels, and is currently approved for injections by the US Food and Drug Administration (FDA) [34].

In addition to the beneficial attributes of natural polysaccharides (biocompatibility, biodegradability, mucoadhesive properties, and low toxicity), hyaluronic acid also exhibits great potential for targeted drug delivery due to the presence of hyaluronic acid receptors in many tissues, such as the liver, kidneys, and most cancer tissues [12,128]. For nucleic acid delivery applications, it is frequently used in combination with cationic polymers because its polyanionic structure enables the neutralization of the positive charge of polycations, reducing their associated toxicity and increasing their stability in physiological media [34].

### 4.1. Factors Affecting Gene Silencing Efficiency and Applications of Native Hyaluronic Acid-Based Nanocarriers

Carbohydrates typically stabilize nucleic acids through hydrogen bonds and Van der Waals interactions [129]. In a recent study, Paidikondala and co-workers proved the capacity of hyaluronic acid to stabilize siRNA by thermal melting experiments, gel mobility shift assay, dynamic light scattering, scanning electron microscopy, as well as computational methods. A systematic analysis of the interactions between HA and siRNA was performed using different sizes of HA polymer, HA:siRNA ratios, and salt concentrations. The authors found that 200 and 300 kDa HA self-assembled with siRNA to form nanoparticles with higher stability than in 100 kDa HA, which was directly related with a higher gene silencing effect. They also demonstrated that the presence of salt was needed to minimize the ionic repulsion between HA and siRNA. By taking these considerations into account, they achieved a 55% gene silencing efficiency *in vitro* with the optimized HA:siRNA ratio [130].

Anionic nanocarriers made solely of siRNA and hyaluronic acid can be prepared by ionic or covalent crosslinking to form nanogels. Forti and co-workers used calcium ions to ionically crosslink the negatively charged siRNA and hyaluronic acid [131]. Their nanoparticles achieved over 80% gene silencing in an *in vitro* model of acute inflammatory response in human hepatocytes. For covalent crosslinking, Lee and co-workers fabricated nanogels with thiol-conjugated hyaluronic acid using an inverse emulsion method followed by crosslinking through disulfide bonds. During the emulsification process, siRNA was physically entrapped with an encapsulation efficiency of ~50% and inhibited 60% of GFP expression in HCT-116 cells [132].

Nevertheless, siRNA nanocarriers based solely on hyaluronic acid are not frequently designed because hyaluronic acid’s negative charge does not favor siRNA loading. Therefore, most hyaluronic acid-based nanocarriers for siRNA delivery incorporate chemical modifications or include a cationic component, usually another polymer, to enhance siRNA loading by electrostatic interactions. The nature of the included polycation or modification deeply influence the whole system’s gene silencing efficiency.

### 4.2. Modifications to Improve the Efficiency of Hyaluronic Acid-Based siRNA Nanocarriers

Unlike cationic polymers such as chitosan, the main shortcoming of hyaluronic acid for siRNA delivery is its low binding capacity for nucleic acids due to electrostatic repulsion, what usually gives rise to poor siRNA loading efficiencies and stability issues. However, these limitations are being surpassed thanks to research into new methods to modify hyaluronic acid or use it as a coating of siRNA–polycation complexes. When hyaluronic acid is exposed on the outer shell, it endows the nanocarrier with its natural targeting ability, which can be further modulated depending on the therapeutic objective. This property is extensively described in the Section 4.2.2 and it is being exploited to improve anticancer treatments by the specific co-delivery of siRNA and chemotherapeutic drugs to the tumour site.

#### 4.2.1. siRNA Loading and Stability

In contrast to the traditional siRNA polyplexes that are obtained by electrostatic interactions, an innovative approach to load siRNA into hyaluronic acid nanocarriers was recently developed by Choi and colleagues [133]. They designed self-assembled nanocarriers formed by amphiphilic hyaluronic acid conjugated to cholesterol, providing a hydrophobic core where 2b RNA-binding protein/siRNA complexes are subsequently entrapped to form a supramolecular assembly of 192 nm. The high affinity of siRNA for 2b protein allows the neutralization of negatively-charged nucleic acid cargo and allows its encapsulation into the nanocarrier core, which also protects siRNA against enzymatic degradation. However, they reached an siRNA loading of only 11% with this modification strategy [133].

Higher siRNA loadings are usually obtained with polyelectrolyte complexes. However, the electrostatic repulsion between siRNA and hyaluronic acid, both negatively charged, prevent the formation of polyelectrolyte complexes, unless a cationic additive is present. The cationic component allows the formation of electrostatic interactions with the siRNA, which usually improves the siRNA loading of the hyaluronic acid-based nanocarrier. On the other hand, hyaluronic acid has been used in association with cationic nanocarriers to enrich them with improved properties such as biocompatibility, reduced cytotoxicity, and specific targeting [134,135].

PEI is clearly one of the most successful cationic polymers used in combination with hyaluronic acid. In fact, nanocarriers combining these two polymers enjoy the advantages that each polymer supplies. The presence of hyaluronic acid reduces the cytotoxicity of PEI-containing nanomaterials [136]. At the same time, branched PEI (bPEI) exercises a “proton sponge effect” in acidic intracellular compartments, which promotes the siRNA’s endosomal escape [137]. There are also a number of publications combining hyaluronic acid with chitosan for siRNA delivery, where chitosan improves the siRNA binding, while hyaluronic acid confers targeting ability and increases the stability of the delivery system (these literature examples and further details about the benefits of the association of these two natural polysaccharides can be found in the Section 5).

Multimerization of siRNAs into polysiRNA through disulfide bonds originates a long siRNA double strand formed by individual siRNA molecules covalently bonded. This has been reported to be an effective strategy to increase the encapsulation efficiency into bPEI/HA polyplexes as well as their stability in the vitreous humor (the extracellular matrix in the center of the eye) to treat retinal or choroidal disorders. Once in the cytosol, where there is a high concentration of glutathione, disulfide bonds are cleaved and lengthened siRNA splits back into single molecules, which makes it easier for siRNA to interact with RISC proteins [138].

siRNA can also be chemically conjugated to hyaluronic acid and further complexed with a polycation. Compared with physical encapsulation, covalent conjugation provides a longer circulation time in plasma and a reduced burst release of the cargo [139]. However, despite the wide application of polymer−drug conjugates, there are only a few polymer−siRNA conjugates currently being investigated, this could be due to the fact that it is essential that siRNA is completely free for the correct formation of a RISC complex. A study by Park and colleagues described that hyaluronic acid conjugated to siRNA by an easily cleavable bond (reducible disulfide bond) and further complexed with linear PEI (LPEI) showed a higher *in vitro* gene silencing efficiency compared to the complex formed with non-cleavable HA−siRNA conjugate and LPEI [140].

The association of hyaluronic acid with cationic polymers to form the nanocarriers for siRNA delivery can be mediated by electrostatic interactions or by covalent interactions. Table 2 compiles the most frequently used cationic polymers that are combined with hyaluronic acid. It also provides information about the type of association between hyaluronic acid and the cationic polymer, the components of the delivery system, the type of nanoparticle formed, the *in vitro* and/or *in vivo* conditions of the studies, and if a combination therapy strategy has been used.

Some properties of siRNA/polycation/hyaluronic acid nanocarriers formed by electrostatic interactions are influenced by the component mixing order during their preparation [141,154]. siRNA protection, cellular uptake, and gene silencing were found to be better when siRNA was initially mixed with hyaluronic acid or hyperbranched poly(amido amine) (PCD) and then added to the oppositely charged polymer, than when HA/PCD binary complexes were prepared and subsequently incubated with siRNA. The authors addressed the results observed to the formation of different assembly structures [141].

According to the literature, the mixing order most commonly used consists of the initial formation of the siRNA/polycation complex followed by a hyaluronic acid coating [136,138,142,143]. However, simple physical assembly based on electrostatic interactions between the shell and the core may undergo to destabilization under *in vivo* conditions if the polycation in the core become deprotonated [155]. Moreover, the anionic shell can be competitively displaced by negatively charged components in the bloodstream, which results in subsequent aggregation of formulations. To solve this issue, Yin and co-workers have used disulfide bonds to chemically cross-link the hyaluronic acid coating around a siRNA/PEI core [142]. This strategy, relying on both electrostatic forces and thiol cross-linking, improved the firmness of the surface shell, thereby enhancing the stability of such nanocarriers both *in vitro* and *in vivo* [142].

**Figure 6:**
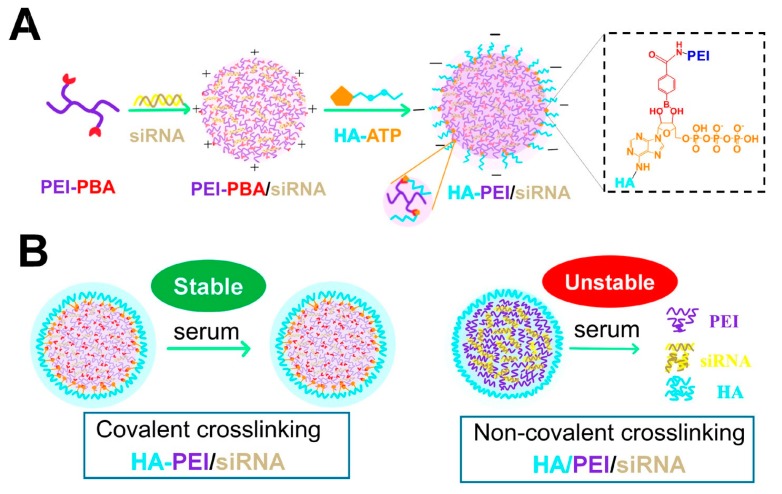
Scheme of the formation of covalent crosslinked polyplex hyaluronic acid (HA)- polyethylenimine (PEI)/siRNA (**A**) and illustration of serum tolerance of covalently crosslinked HA-PEI/siRNA versus poor serum stability of non-covalently crosslinked HA-PEI/siRNA (**B**). Reproduced from [150] with permission from American Chemical Society.

While some researchers prefer to associate hyaluronic acid to polycations by electrostatic complexation on the argument that chemical modification could disturb the functionalities of native hyaluronic acid [156], others prefer to chemically modify hyaluronic acid by conjugating it to cationic groups or polymers that confer the positive charge needed for siRNA complexation [157]. This covalent attachment could result in a more stable and permanent availability of hyaluronic acid in siRNA nanocarrier compared to pH-dependent electrostatic complexation [32]. Zhou and co-workers demonstrated the superior colloidal stability of the covalent conjugate HA-PEI in the presence of serum and phosphate in comparison to non-covalent polyplex HA/PEI [150]. Moreover, they used a reversible covalent crosslinking that allowed the controlled release of siRNA in the ATP-rich environment of cytosol and significantly improve siRNA silencing (Figure 6). Note that in this review, following the standard nomenclature used in literature, covalent linking between HA and a polycation is referred as HA-polycation and non-covalent linking is denoted as HA/polycation.

The combination of HA-PEI with HA-PEG conjugates to form siRNA nanocarriers is giving promising *in vivo* results. In fact, among an extensive screening of functionalized hyaluronic acid derivatives to form self-assembling nanosystems with siRNAs, HA-PEI/HA-PEG/siRNA was found to be the ideal system that could deliver the siRNA most efficiently and showed the highest target gene knockdown in subcutaneous A549 tumours [147].

Apart from PEI, other polycations have also been conjugated to hyaluronic acid to form nanosized siRNA delivery systems and have shown efficient *in vitro* or *in vivo* gene silencing, such as spermine (a natural tetraamine) [152] and poly(dimethylaminoethyl methacrylate) (pDMAEMA) [153], respectively.

#### 4.2.2. Cell Targeting Specificity and Biodistribution

The great potential of hyaluronic acid for targeted drug delivery derives from the presence of hyaluronic acid receptors in many tissues (i.e., liver, kidneys, and most cancer tissues) [128,158,159]. There are several proteins identified as hyaluronic acid receptors: CD168 (also called RHAMM, receptor for hyaluronate-mediated motility) and CD44 are usually overexpressed in cancerous cells; LYVE-1 is the lymphatic vessel endothelial hyaluronic acid receptor-1 [127]; and HARE is the hyaluronic acid receptor for endocytosis, abundantly expressed in the sinusoidal endothelial cells of the liver [160].

Consequently, hyaluronic acid-based nanocarriers are an excellent system for the specific delivery into the liver and they have been used to treat liver-related diseases [144,161]. In a recent study, *in vivo* fluorescence showed that HA-PEI/siRNA nanopolyplexes significantly accumulated in the liver, while PEI/siRNA mainly accumulated in the lung. This targeted siRNA delivery into cirrhotic liver sinusoidal endothelial cells (LSECs) resulted in highly effective downregulation of COX-1 (an enzyme overexpressed in cirrhosis) and therapeutic improvement, although liver fibrosis was not reversed [162].

Nevertheless, when the liver is not the delivery target, the preferential accumulation of these carriers in the liver, after systemic administration, could be a problem. However, this issue can be solved by the incorporation of chemical modifications to the nanocarrier, such as PEG, which can reduce the *in vivo* liver uptake and increase their circulation time in the blood [147,148,163].

It has been shown that the degree of hyaluronic acid chemical modifications and hyaluronic acid molecular weight are critical factors that influence biodistribution, since they affect the binding affinity between the polysaccharide and its receptors [126]. Mizrahy and co-workers found that low molecular weight hyaluronic acid presented extremely low affinity for receptor CD44, whereas the high molecular weight polymer bound with high affinity [164].

**Figure 7:**
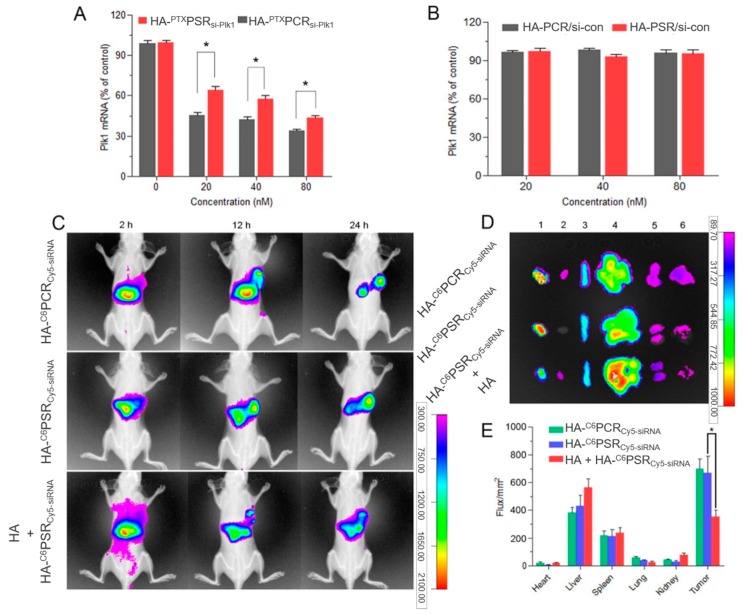
*In vivo* siRNA delivery efficiency of adelivery system with a redox-sensitive cationic inner core and detachable hyaluronic acid (HA) crosslinked outer shell, named as HA-PSR, for simultaneous delivery of siRNA and paclitaxel (PTX) within tumour cells. HA-PCR corresponds to a version of the delivery system with a non-redox-sensitive core. Gene silencing was assessed through mRNA quantification and biodistribution was analyzed using fluorescently labeled siRNA (Cy5-siRNA) loaded into fluorescently labeled nanoparticles (HA-^C6^PCR or HA-^C6^PSR). Plk1 mRNA expression at different si-Plk1 concentrations (**A**) or scrambled siRNA (si-con) concentrations (**B**) determined by qRT-PCR. (**C**) *In vivo* fluorescent imaging of A549 tumour-bearing nude mice after intravenous injection of C6/Cy5-siRNA co-loaded HA-PSR/HA-PCR nanoparticles with or without pre-injection of free HA. (**D**) Ex vivo Cy5 fluorescence imaging of the tumour and normal tissues of the A549 tumour-bearing nude mice at 24 h post-injection. 1, tumour; 2, heart; 3, spleen; 4, liver; 5, kidney; and 6, lung. (**E**) Region-of-interest (ROI) analysis of Cy5 fluorescent signals from the tumours and normal tissues. *p < 0.05. Adapted from [142] with permission from Elsevier.

CD44-overexpressing cancers are exceptional targets for the selective delivery of siRNA using hyaluronic acid-based nanocarriers. A preferential accumulation of fluorescently labeled siRNA loaded into fluorescently labeled HA nanoparticles was observed by Yin and co-workers in tumour site after 24 h. Moreover, when mice were pre-administered with a dose of free HA to saturate CD44 receptors, the fluorescence signal in the tumour significantly weakened compared to that of unsaturated receptors (Figure 7), demonstrating the role of HA in active targeting through CD44-mediated endocytosis [142].

#### 4.2.3. Co-Delivery of siRNA and Pharmaceuticals

There are several examples of siRNA and drug co-delivery using hyaluronic acid/polycation/siRNA complexes, mainly addressed to improve anticancer treatments. Conventional chemotherapy agents exhibit strong hydrophobicity. Therefore, in most cases the nanocarrier system has been modified to have a hydrophobic core where the hydrophobic drug is entrapped, either through inclusion of hydrophobic polymers as poly(lactic-co-glycolic acid) (PLGA) [136,143,165] or polycation hydrophobic derivatizations as octyl [142] and octandioic acid-modified [146] PEI. The hydrophobic core is surrounded by a cationic layer where the siRNA is electrostatically loaded and finally covered by an external negative shell of hyaluronic acid that provides both active targeting and protection from undesired drug release during circulation [136,142,143]. However, there are also some hydrophilic drugs, such as epigallocatechin-3-*O*-gallate (EGCG), that can directly interact with the siRNA, polycation, and hyaluronic acid, avoiding the need of a hydrophobic loading microenvironment in the nanocarrier [145].

**Figure 8:**
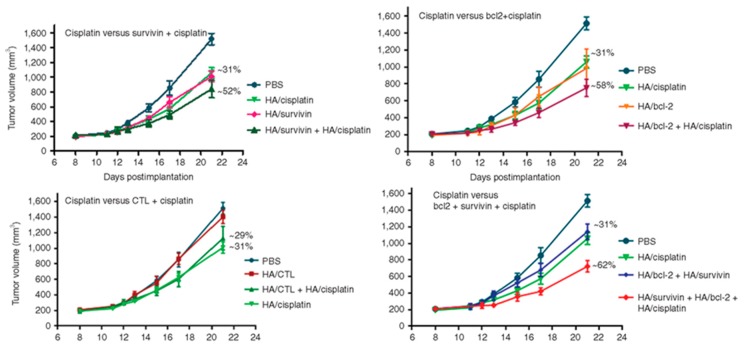
Effect of the combination of the downregulation of two antiapoptotic genes (survivin and bcl-2) and cisplatin treatment. Comparison of the antitumor efficacy of cisplatin-treated mice with survivin + cisplatin-treated mice, cisplatin-treated mice with bcl-2 + cisplatin-treated mice, cisplatin-treated mice with CTL siRNA (non-targeting control siRNA) + cisplatin-treated mice, cisplatin-treated mice with survivin + cisplatin, and survivin + bcl-2 + cisplatin-treated mice. Reproduced [149] from with permission from Elsevier.

Although co-delivery of siRNA and drug together in the same nanocarrier may maximize their therapeutic efficacy, the sequential delivery of siRNA and drug has also demonstrated therapeutic benefits. A synergistic antitumour response in cisplatin resistant tumours was induced by the tandem delivery of siRNA loaded hyaluronic acid nanoparticles, which downregulated genes responsible for cisplatin resistance, followed by treatment with cisplatin loaded hyaluronic acid nanoparticles. Although the resistance was still not completely reversed (the inhibition of the tumour growth was increased from 30 to 60%), it may be due to the involvement of other genes in the resistance mechanism (Figure 8) [148,149]. In a similar study, HA-PEI/HA-PEG/MDR1 siRNA nanoparticle therapy increased cell sensitivity to paclitaxel in multidrug resistance (MDR) ovarian cancer mouse models, and the subsequent paclitaxel treatment inhibited tumour growth [166].

## 5. siRNA Nanocarriers Combining Chitosan and Hyaluronic Acid

siRNA/chitosan binary complexes offer many advantages, such as high siRNA binding ability and rapid cellular internalization (further described in sections of this review related to chitosan-based nanocarriers). However, these complexes are prone to aggregation in the presence of serum proteins, are rapidly removed by the mononuclear phagocyte system, and can also induce systemic toxicity during intravenous administration [141,142]. On the other hand, siRNA/hyaluronic acid binary complexes are harder to construct because of the electrostatic repulsion between both negatively charged macromolecules, leading to low siRNA loading efficiency and stability issues. The association of siRNA with both chitosan polycation and hyaluronic acid polyanion is an effective strategy to overcome these problems, where chitosan ensures a strengthened siRNA binding and promotes endosomal escape, while the hyaluronic acid provides stability, low protein adsorption, and an inherent capacity to target liver and CD44 overexpressing tumours [34,167].

The formation of chitosan matrices cross-linked with TPP by ionotropic gelation, inside which siRNA and hyaluronic acid molecules are condensed, has been reported. As depicted in this interesting study, using isothermal titration calorimetry, hyaluronic acid competed with siRNA for chitosan binding, lowering chitosan–siRNA binding strength by 25%. This suggests that besides improving cell biocompatibility of chitosan, hyaluronic acid might also promote siRNA release by loosening the chitosan–siRNA binding [134]. In other works, chitosan/siRNA polyplexes have been coated with hyaluronic acid to increase the targeting of CD44^+^ tumor endothelial cells (by 2.1-fold) [168] and to improve haemocompatibility for intravenous administration [169].

A comparative *in vitro* analysis between two HA-based polyplex systems (one including poly(hexamethylene biguanide) and the other one chitosan as the cationic component) revealed that chitosan nanoparticles present the right compromise between polyplex stability and siRNA release. The stronger interactions between poly(hexamethylene biguanide) and siRNA (and HA) negatively affected the silencing efficacy of such nanoparticle, possibly due to the lower availability of the nucleic acid, although they provided a higher stability against nucleases [167].

## 6. Conclusions

The therapeutic success of siRNA-based formulations hinges on overcoming the many challenges involved with siRNA delivery *in vivo.* The development of siRNA delivery systems that are simultaneously safe and effective will be decisive for the successful application of siRNA-based therapies. Nanostructured delivery systems provide unique advantages, such as protection from premature degradation and improved interaction with the biological environment. They also offer the possibility to enhance tissue absorption, extend siRNA residence time, improve cellular internalization, and facilitate siRNA transport into the cytoplasm of target cells.

In this review we focused on siRNA delivery systems based on two natural polysaccharides: chitosan and hyaluronic acid, which are widely studied since they are commonly considered to be highly biocompatible, biodegradable, and non-immunogenic mucoadhesive polymers. On the one hand, positively charged chitosan can interact with siRNA to form stable polyplexes. Such strong electrostatic interactions help protect the nucleic acid from degradation but may also limit its release. The main hurdles associated with the clinical use of chitosan-based delivery systems are their low stability at physiological pH, weak buffering capacity, and lack of cell specificity. However, understanding these limitations has encouraged the use of chitosan derivatives as alternative delivery systems to combat the aforementioned deficiencies. On the other hand, anionic polysaccharides, such as hyaluronic acid, often require the presence of cationic components to favor a more efficient interaction with siRNA. However, hyaluronic acid has displayed great potential for the specific delivery of siRNA to tissues such as the liver and tumours, which express its natural receptors. Furthermore, the association of siRNA with both chitosan and hyaluronic acid is an effective strategy whereby chitosan ensures a strengthened siRNA binding while hyaluronic acid provides stability, low protein adsorption, and targeting capacity.

The promising results obtained *in vitro* and *in vivo* with both biopolymers has shown their potential as siRNA delivery systems for gene silencing applications and the development of multifunctional co-delivery systems, especially those combining siRNA and chemotherapy drugs represent particularly promising synergistic anticancer treatments [86,115,142,149]. However, more effective and non-toxic nanocarriers based on these polysaccharides that could one day reach clinical trials are still a long way off. Future work should focus on the design of the nanocarrier by exploring chemical modifications that have not been tested yet and by combining the advantages of different polymeric materials to obtain highly efficient and biocompatible hybrid nanosystems.

## Figures and Tables

**Table 1:** Literature examples of chitosan modifications for small interfering RNA (siRNA) delivery.

Components of the Delivery System	CS Modifications	Improvement of the Modification	*In vitro*/*In vivo* Study	Combination Therapy	Ref
Delivery system 1. Non-target delivery: CS, PEG, siRNA Delivery system 2. Target delivery: CS, PEG, EGFR-targeting peptide, siRNA	PEG EGFR peptide	Colloidal stability and longer half-life Target EGF receptor (EGFR)	*In vitro* + *in vivo*	Sequential delivery: 1. NPs siRNA 2. cisplatin	[78,79]
CS, PEG, siRNA	PEG	Solubility and stability	*In vitro* + *in vivo*	-	[80]
N-succinyl-CS, doxorubicin, poly-l-lysine (PLL), palmitic acid, siRNA	Succinyl PLL Palmitic acid	Solubility siRNA binding Hydrophobic core	*In vitro* + *in vivo*	Co-delivery: siRNA doxorubicin	[81]
CS, PEI, TPP, PEG, RGDp, siRNA	PEG RGDp	Solubility and stability Target α_v_β_3_ integrin receptors	*In vitro* + *in vivo*	Co-delivery: 2 siRNAs	[82]
N-succinyl CS, paclitaxel, lipoic acid (LA), low-density lipoprotein (LDL), cholesterol-siRNA	Succinyl LA LDL Cholesterol	Solubility Hydrophobic core Target LDL receptor Hydrophobic interactions with LA	*In vitro* + *in vivo*	Co-delivery: siRNA paclitaxel	[83]
Low Molecular weight (LMw) CS, protamine, TPP, siRNA	LMw CS Protamine	Solubility and colloidal stability siRNA binding and cell uptake	*In vitro* + *in vivo*	-	[77]
Trimethyl-CS, cysteine, mannose, siRNA	Trimethyl Cysteine Mannose	Solubility Stability (disulphide bonds) Target enterocites and M cells (intestinal absorption) and macrophages	*In vitro* + *in vivo*	-	[84]
Glycol-CS, sulfosuccinimidyl 6-[3′(2-pyridyldithio)-propionamido] hexanoate (Sulfo-LC-SPDP), dual-poly-siRNA	Glycol Sulfo-LC-SPDP	Solubility Stability (disulphide bonds)	*In vitro* + *in vivo*	Co-delivery: 2 siRNAs	[85]
Delivery system 1. siRNA-loaded: Glycol-CS, sulfosuccinimidyl 6-[3′(2-pyridyldithio)-propionamido] hexanoate (Sulfo-LC-SPDP), siRNA Delivery system 2. Doxorubicin-loaded: Glycol-CS, 5β-cholanic acid, doxorubicin	Glycol Sulfo-LC-SPDP Cholanic acid	Solubility Stability (disulphide bonds) Hydrophobic core	*In vitro* + *in vivo*	Sequential delivery: 1. NPs doxorubicin 2. NPs siRNA	[86]
CS, TAT, siRNA	TAT	Cell uptake	*In vitro* + *in vivo*	-	[87]
CS, poly(histidine-arginine)_6_ (H6R6) peptide, siRNA	H6R6	Cell uptake and endosomal escape	*In vitro* + *in vivo*	-	[88]
CS, antibody, siRNA	IgG antibody	Target M1 macrophages	*In vitro* + *in vivo*	-	[89]
Trimethyl CS, PEG, mannose, poly-(allylamine hydro- chloride)-citraconic anhydride (PC)	Trimethyl PEG Mannose PC	Solubility Colloidal stability and longer half-life in blood Target macrophages Endosomal escape	*In vitro* + *in vivo*	Co-delivery: 2 siRNAs	[90]
CS, PEG, folic acid and diethylethylamine (DEAE)	PEG Folic acid DEAE	Linker Target activated macrophages Colloidal stability	*In vitro* + *in vivo*	-	[91]
Delivery system 1: Trimethyl-CS, 3 siRNAs Delivery system 2: Imidazole-CS, 3 siRNAs	Trimethyl Imidazole	Solubility and enhanced mucoadhesive properties Endosomal escape	*In vitro*	Co-delivery: 3 siRNAs	[92]
CS, carboxymethyl dextran (CMD), doxorubicin, siRNA	CMD	Colloidal stability and longer half-life	*In vitro*	Co-delivery: siRNA doxorubicin	[93,94]
CS, carboxymethyl dextran (CMD), SN38, siRNA	CMD	Colloidal stability and longer half-life	*In vitro*	Co-delivery: siRNA SN38	[95]
CS, cholesterol, curcumin, siRNA	Cholesterol	Hydrophobic core	*In vitro*	Co-delivery: siRNA curcumin	[96]
CS-lactate, TPP, siRNA	Lactate	Solubility	*In vitro*	-	[97]
Delivery system 1. Without modification: CS, siRNA Delivery system 2. With modification: CS, PEG, siRNA	PEG	Colloidal stability and longer half-life	*In vitro*	-	[98]
LMw O-carboxymethyl-CS, bPEI, human epidermal growth factor receptor 2 (HER-2/neu), siRNA	LMw CS O-carboxymethyl bPEI HER-2/neu	Solubility and colloidal stability Solubility Cell uptake and endosomal escape Targeting ligand	*In vitro*	-	[99]
CS, PEG, siRNA	PEG	Solubility and stability	*In vitro*	-	[100]
CS, urocanic acid, siRNA	urocanic acid	Endosomal escape and siRNA binding	*In vitro*	-	[101]
CS, PEG, PEI, RGD, siRNA	PEG PEI RGD	Solubility and stability Cell uptake and endosomal escape Target α_v_β_3_ integrin receptors	*In vitro*	-	[102]
CS, PEG, HA, PEI, TPP, siRNA	PEG HA PEI	Solubility and stability Stability Cell uptake and endosomal escape	*In vitro*	-	[103]
Trimethyl CS, cysteine, mannose	Trimethyl Cysteine Mannose	Intestinal mucoadhesion Intestinal mucoadhesion Target enterocites and M cells (intestinal absorption)	*In vitro*	-	[104]
CS, PEG, TAT, siRNA	PEG TAT	Linker between CS and TAT Cell uptake	*In vitro*	-	[105]
CS, nonaarginine, siRNA	nonaarginine	Cell uptake	*In vitro*	-	[106]
CS, PEI, CMD	PEI CMD	Endosomal escape Colloidal stability	*In vitro*	-	[17]

CS: chitosan, PEG: polyethylene glycol, siRNA: small interfering RNA, EGF: epidermal growth factor, EGFR: EGF receptor, NPs: nanoparticles, PLL: poly-l-lysine, PEI: polyethylenimine, bPEI: branched PEI, TPP: tripolyphosphate, RGD: Arg-Gly-Asp tripeptide, RGDp: RGD peptidomimetic, LA: lipoic acid, LDL: low-density lipoprotein, LMw: Low Molecular weight, Sulfo-LC-SPDP: sulfosuccinimidyl 6-[3′(2-pyridyldithio)-propionamido] hexanoate, TAT: Trans Activator of Transcription peptide, H6R6: poly(histidine-arginine)_6_, IgG: Immunoglobulin G, PC: poly-(allylamine hydro- chloride)-citraconic anhydride, DEAE: diethylethylamine, CMD: carboxymethyl dextran, HER-2/neu: human epidermal growth factor receptor 2, HA: hyaluronic acid.

**Table 2:** Literature examples of hyaluronic acid association with cationic polymers for siRNA delivery.

Type of Association between Hyaluronic Acid and Cationic Polymer	Cationic Polymer	Components of the Delivery System	Type of Nanoparticle	*In vitro*/*In vivo* Study	Combination Therapy	Ref
Electrostatic interaction	PCD	HA, hyperbranched poly(amido amine) (PCD), siRNA	Polyelectrolyte complex	*In vitro*	-	[141]
	PEI	HA-cystamine, Octyl-ss-PEI (reducible bond) or Octyl-cc-PEI (non-reducible bond), paclitaxel, siRNA	Polymeric micelle	*In vitro* + *in vivo*	Co-delivery: siRNA paclitaxel	[142]
	PEI	HA, PLGA-ss-PEI (reducible bond) or PLGA-PEI (non-reducible bond), docetaxel, siRNA	Polymeric micelle	*In vitro* + *in vivo*	Co-delivery: siRNA docetaxel	[143]
	LPEI	HA-ss-siRNA (reducible bond) or HA-siRNA (non-reducible bond), LPEI	Polyelectrolyte complex	*In vitro* + *in vivo*	-	[140]
	bPEI	HA, bPEI, polysiRNA	Polyelectrolyte complex	*In vitro* + *in vivo*	-	[138]
	CS	HA, CS, TPP, siRNA	Nanogel	*In vitro*	-	[134]
	Protamine	HA-taurocholic acid, protamine, siRNA	Polyelectrolyte complex	*In vitro* + *in vivo*	-	[144]
	Protamine	HA, Protamine, epigallocatechin-3-O-gallate (EGCG), CPP, siRNA	Nanogel	*In vitro* + *in vivo*	Co-delivery: siRNA EGCG	[145]
Covalent linking	bPEI	HA-ss-(Octandioic acid-g-bPEI) or HA-(Octandioic acid-g-bPEI), paclitaxel, siRNA	Polymeric micelle	*In vitro* + *in vivo*	Co-delivery: siRNA paclitaxel	[146]
	bPEI	HA-bPEI, HA-PEG, HA-Hexyl fatty acid, Cholesterol-siRNA	Polymeric micelle	*In vitro* + *in vivo*	-	[137]
	bPEI	Selected system after HA derivatives screening: HA-PEI, HA-PEG, siRNA or cisplatin Other HA-derivatives studied: HA-butylamine, HA-hexylamine, HA-octylamine, HA-stearylamine, HA-1,6 diaminohexane, HA-1,8 diaminooctane, HA-choline, HA-spermine,	Polyelectrolyte complex	*In vitro* + *in vivo*	Sequential delivery: 1. NPs siRNA 2. NPs cisplatin	[147,148,149]
	PEI	HA-PEI, siRNA	Polyelectrolyte complex	*In vitro* + *in vivo*	-	[150]
	PEI	HA-PEI, heparin, Ca2+, siRNA	Polyelectrolyte complex	*In vitro*	-	[151]
	Spermine	HA-spermine, siRNA	Polymeric micelle	*In vitro*	-	[152]
	pDMAEMA	HA-pDMAEMA- 2-(2-pyridyldithio) ethylamine (PDA), siRNA	Polyelectrolyte complex	*In vitro* + *in vivo*	-	[153]
